# Supplementary material for: Evolution of sex‐biased genes in Drosophila species with neo‐sex chromosomes: Potential contribution to reducing the sexual conflict
Source: Ecol Evol. 2024 Jul 23;14(7):e11701. doi: 10.1002/ece3.11701 (PMC11266434; doi:10.1002/ece3.11701)
Supplement: Supplementary file 1 — Data S1: [file ECE3-14-e11701-s001.zip › Supp_figures240618.pdf]

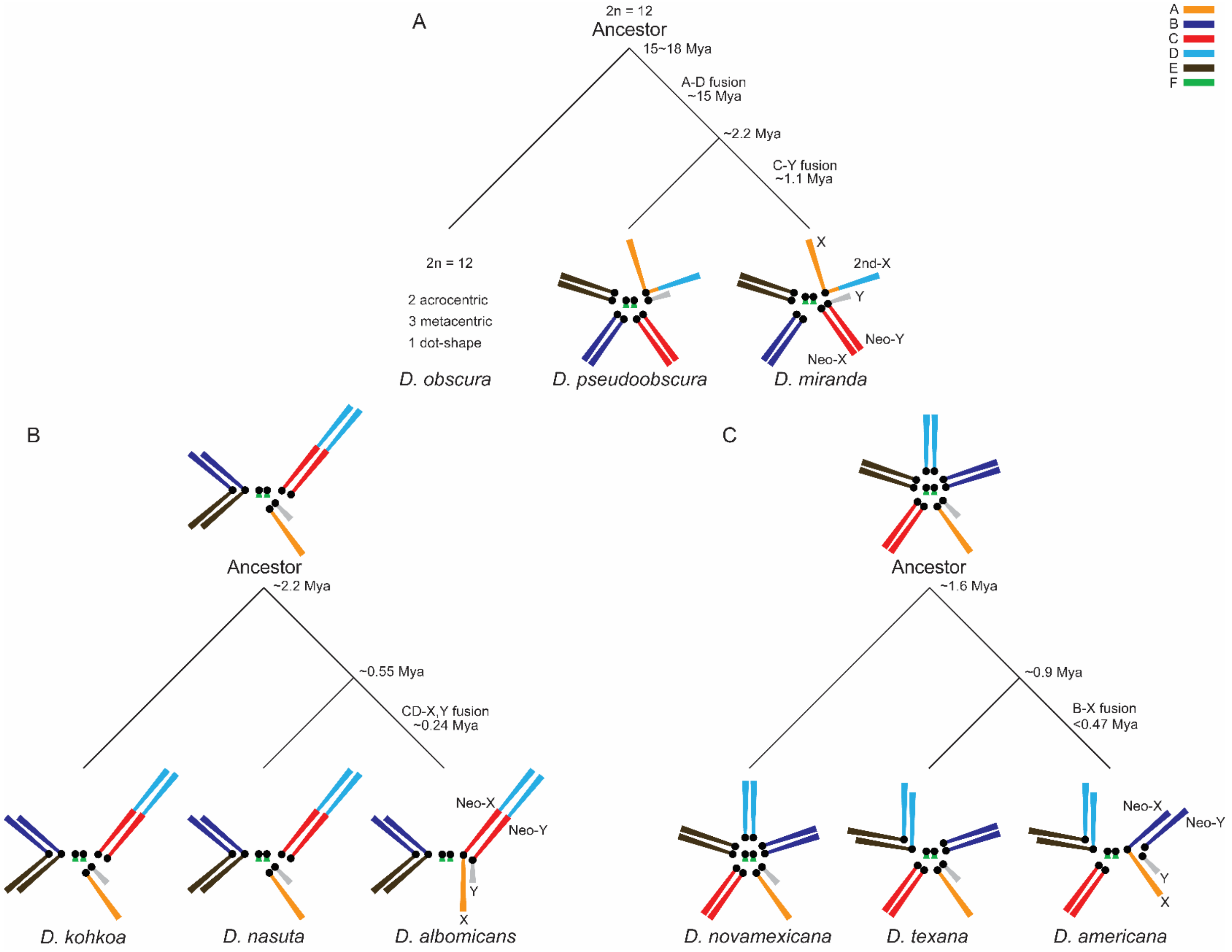
A
2n = 12
Ancestor
15~18 Mya
A-D fusion
~15 Mya
~2.2 Mya
C-Y fusion
~1.1 Mya
2n = 12
2 acrocentric
3 metacentric
1 dot-shape
X
2nd-X
Y
Neo-Y
Neo-X
D. obscura
D. pseudoobscura
D. miranda
A
B
C
D
E
F
B
Ancestor
~2.2 Mya
~0.55 Mya
CD-X,Y fusion
~0.24 Mya
Neo-X
Neo-Y
Y
X
D. kohkoa
D. nasuta
D. albomicans
C
Ancestor
~1.6 Mya
~0.9 Mya
B-X fusion
<0.47 Mya
Neo-X
Neo-Y
Y
X
D. novamexicana
D. texana
D. americana

Figure S1. Phylogenetic relationship of three trio *Drosophila* species, (A) *D. miranda*, *D. pseudoobscura*, and *D. obscura*, (B) *D. albomicans*, *D. nasuta*, *D. kohkoa*, and (C) *D. americana*, *D. texana*, and *D. novamexicana*, with male karyotypes. For karyotypes, colors correspond to the Muller elements (i.e., homologous chromosomes) shown on the top-right. For *D. obscura*, only the number of chromosomes with chromosome types is shown because the correspondence between Muller elements and chromosome shapes remains unclear. Divergence times among *D. miranda*, *D. pseudoobscura*, and *D. obscura* are based on Bachtrog and Charlesworth (2002) and Gao et al. (2007), whereas those among *D. albomicans*, *D. nasuta*, and *D. kohkoa* were retrieved from Nagaraja et al. (2004) and Satomura and Tamura (2016). Divergence times among *D. americana*, *D. texana*, and *D. novamexicana* are from Morales-Hojas et al. (2011). Emergence times of neo-sex chromosomes for *D. miranda*, *D. albomicans*, and *D. americana* were estimated by Bachtrog and Charlesworth (2002), Satomura and Tamura (2016), and Vieira et al. (2003), respectively.

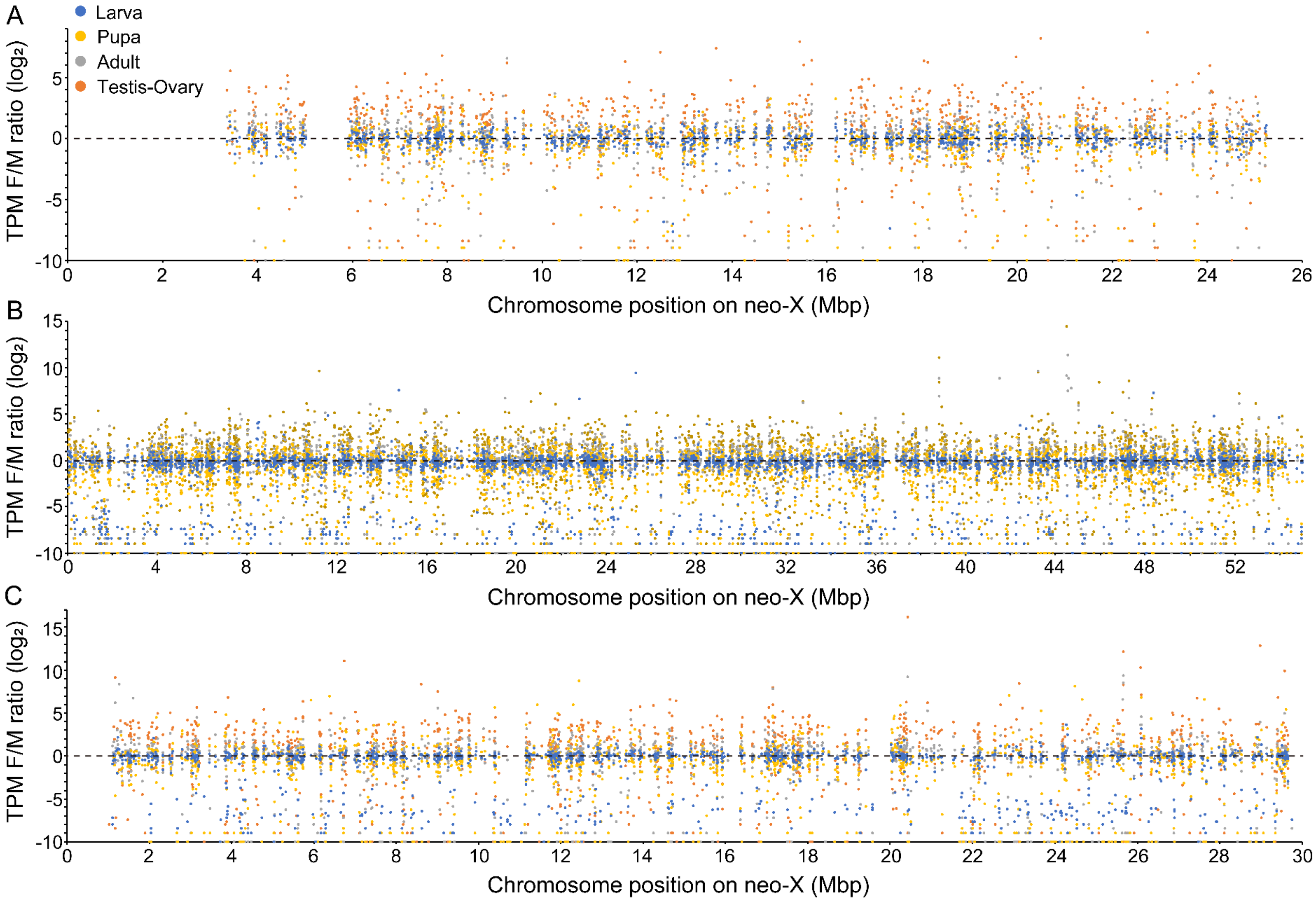
A
Larva
Pupa
Adult
Testis-Ovary
TPM F/M ratio (log2)
Chromosome position on neo-X (Mbp)
B
TPM F/M ratio (log2)
Chromosome position on neo-X (Mbp)
C
TPM F/M ratio (log2)
Chromosome position on neo-X (Mbp)

Figure S2. Relationship between sex-biased genes and their chromosomal positions on neo-X of (A) *D. miranda*, (B) *D. albomicans*, and (C) *D. americana*. The ratio of female to male TPM values was used as an indicator of sex-biased expression. For computing the ratio, we added 0.01 for both numerator and denominator to avoid any inapplicable case. Each dot indicates the chromosomal position on the X axis and the ratio of female to male TPM values (blue, yellow, grey, and orange correspond to larvae, pupae, adults, and gonads, respectively) on the Y axis for each gene. Broken lines represent the ratio of 1 (e.g., unbiased expression). The genes that have orthologs on the same Muller element in the closely related species (e.g., *D. pseudoobscura* and *D. obscura* for *D. miranda*) and show the TPM value of ≥1 in at least either female or male tissues were analyzed.

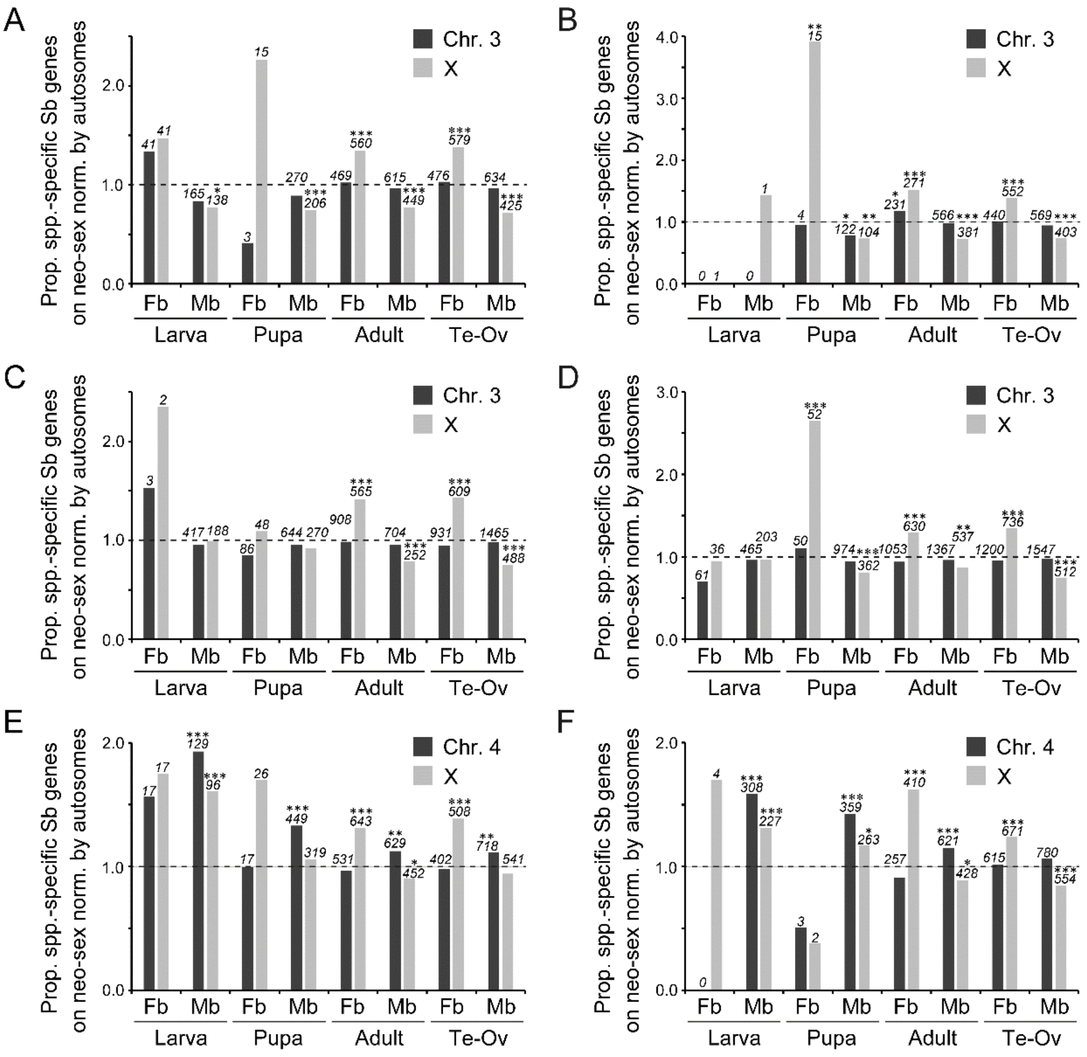

Figure S3. Proportion of sex-biased genes on the autosomes homologous to the neo-sex chromosomes and the X chromosome in (A) *Drosophila pseudoobscura*, (B) *D. obscura*, (C) *D. nasuta*, (D) *D. kohkoa*, (E) *D. texana*, and (F) *D. novamexicana* normalized by the proportion of sex-biased genes on autosomes. A value of 1.0 shown by a broken line indicates that the proportion of sex-biased genes is equal on the target chromosome homologous to the neo-sex chromosomes and autosomes. The numbers of sex-biased genes on the target or the X chromosome are indicated in italics above each bar. Differences between the proportion of sex-biased genes on the target chromosome and autosomes were tested by the Fisher's exact test with correction for multiple testing by the Benjamini-Hochberg method (Benjamini & Hochberg, 1995). *** Q <0.001; ** Q <0.01; * Q <0.05. Sb, sex-biased (either female- or male-biased); Fb, female-biased; Mb, male-biased; Te, testis; Ov, ovary. See Figure S1 for the results for their closely related species.

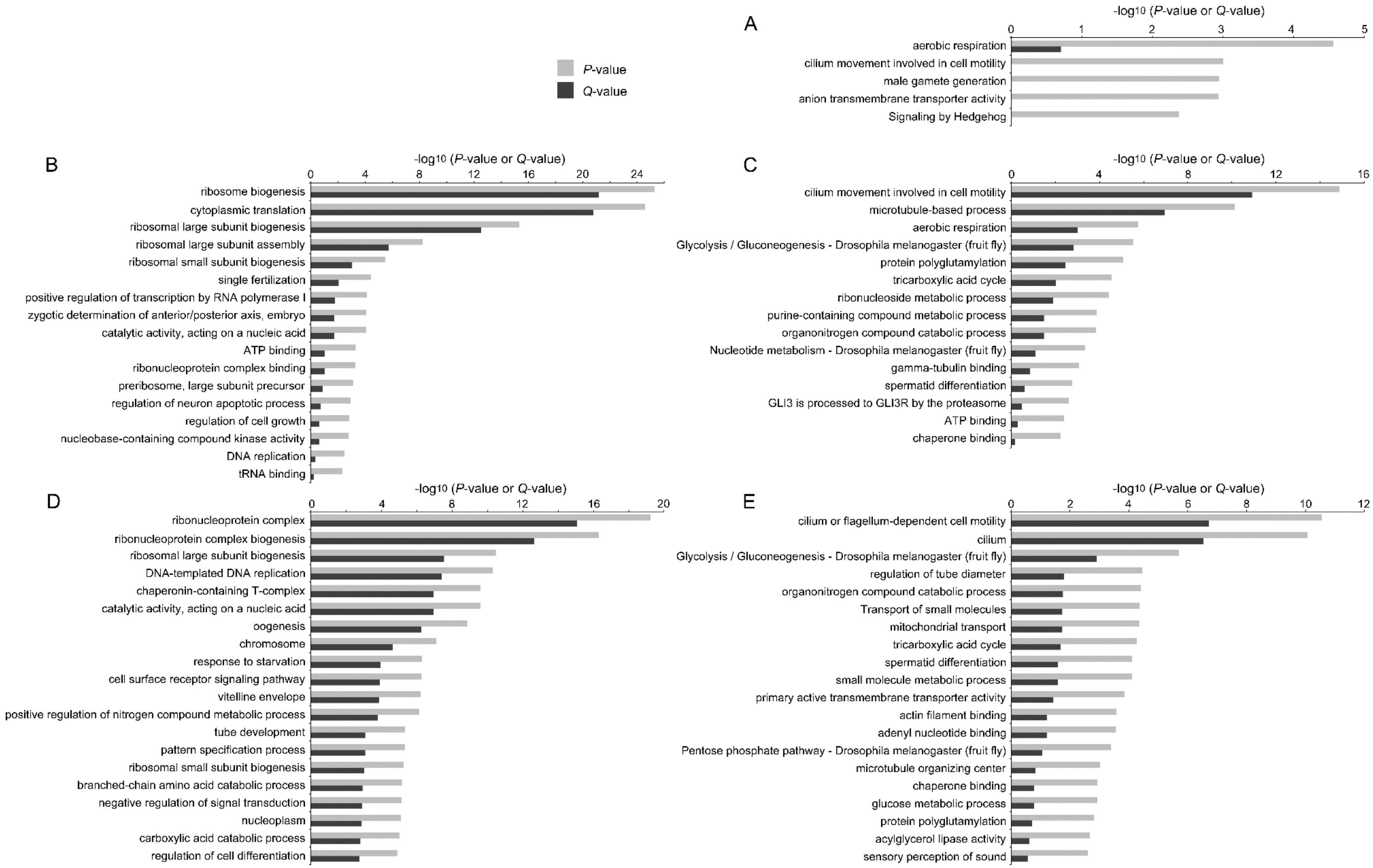
P-value
Q-value
A
-log10 (P-value or Q-value)
aerobic respiration
cilium movement involved in cell motility
male gamete generation
anion transmembrane transporter activity
Signaling by Hedgehog
B
ribosome biogenesis
cytoplasmic translation
ribosomal large subunit biogenesis
ribosomal large subunit assembly
ribosomal small subunit biogenesis
single fertilization
positive regulation of transcription by RNA polymerase I
zygotic determination of anterior/posterior axis, embryo
catalytic activity, acting on a nucleic acid
ATP binding
ribonucleoprotein complex binding
preribosome, large subunit precursor
regulation of neuron apoptotic process
regulation of cell growth
nucleobase-containing compound kinase activity
DNA replication
tRNA binding
C
cilium movement involved in cell motility
microtubule-based process
aerobic respiration
Glycolysis / Gluconeogenesis - Drosophila melanogaster (fruit fly)
protein polyglutamylation
tricarboxylic acid cycle
ribonucleoside metabolic process
purine-containing compound metabolic process
organonitrogen compound catabolic process
Nucleotide metabolism - Drosophila melanogaster (fruit fly)
gamma-tubulin binding
spermatid differentiation
GLI3 is processed to GLI3R by the proteasome
ATP binding
chaperone binding
D
ribonucleoprotein complex
ribonucleoprotein complex biogenesis
ribosomal large subunit biogenesis
DNA-templated DNA replication
chaperonin-containing T-complex
catalytic activity, acting on a nucleic acid
oogenesis
chromosome
response to starvation
cell surface receptor signaling pathway
vitelline envelope
positive regulation of nitrogen compound metabolic process
tube development
pattern specification process
ribosomal small subunit biogenesis
branched-chain amino acid catabolic process
negative regulation of signal transduction
nucleoplasm
carboxylic acid catabolic process
regulation of cell differentiation
E
cilium or flagellum-dependent cell motility
cilium
Glycolysis / Gluconeogenesis - Drosophila melanogaster (fruit fly)
regulation of tube diameter
organonitrogen compound catabolic process
Transport of small molecules
mitochondrial transport
tricarboxylic acid cycle
spermatid differentiation
small molecule metabolic process
primary active transmembrane transporter activity
actin filament binding
adenyl nucleotide binding
Pentose phosphate pathway - Drosophila melanogaster (fruit fly)
microtubule organizing center
chaperone binding
glucose metabolic process
protein polyglutamylation
acylglycerol lipase activity
sensory perception of sound

Figure S4. Terms enriched in (A) shared male-biased genes in pupae, (B) shared female-biased genes in adults, (C) shared male-biased genes in adults, (D) shared female-biased genes in gonads, and (E) shared male-biased genes in gonads among the nine *Drosophila* species examined. Only top 20 terms (P-value < 0.01) are shown at maximum. Metascape software (Zhou et al., 2019) was used for the analysis.

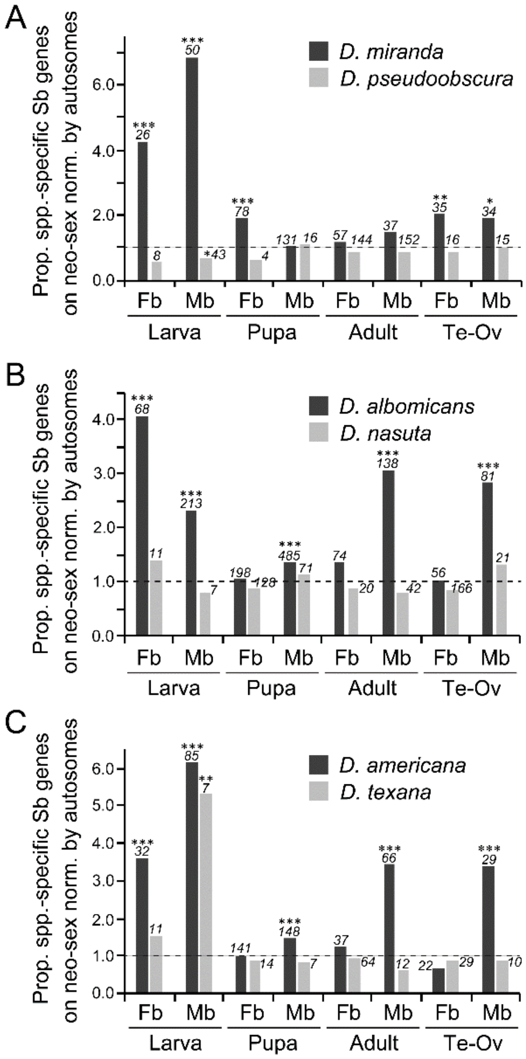

Figure S5. Proportion of genes that acquired sex-biased expression on the neo-sex chromosomes (or the orthologous autosome) in the lineages of (A) *Drosophila miranda* and *D. pseudoobscura*, (B) *D. albomicans* and *D. nasuta*, and (C) *D. americana* and *D. texana* normalized by the proportion of those genes on autosomes. The gene was regarded as female-biased or male-biased if the ratio of female to male TPM values was ≥2 or ≤0.5, respectively. A value of 1.0 by a broken line indicates that the proportion of genes acquiring sex-biased expression is equal in the neo-sex chromosomes (or the orthologous autosome) and autosomes. The numbers of such genes on the neo-sex chromosomes (or the orthologous autosome) are indicated in italics above each bar. Differences between the proportions on the neo-sex chromosomes (or the orthologous autosome) and autosomes were tested by the Fisher's exact test with correction for multiple testing by the Benjamini-Hochberg method (Benjamini & Hochberg, 1995). *** Q <0.001; ** Q <0.01; * Q <0.05. Sb, sex-biased (either female- or male-biased); Fb, female-biased; Mb, male-biased; Te, testis; Ov, ovary.

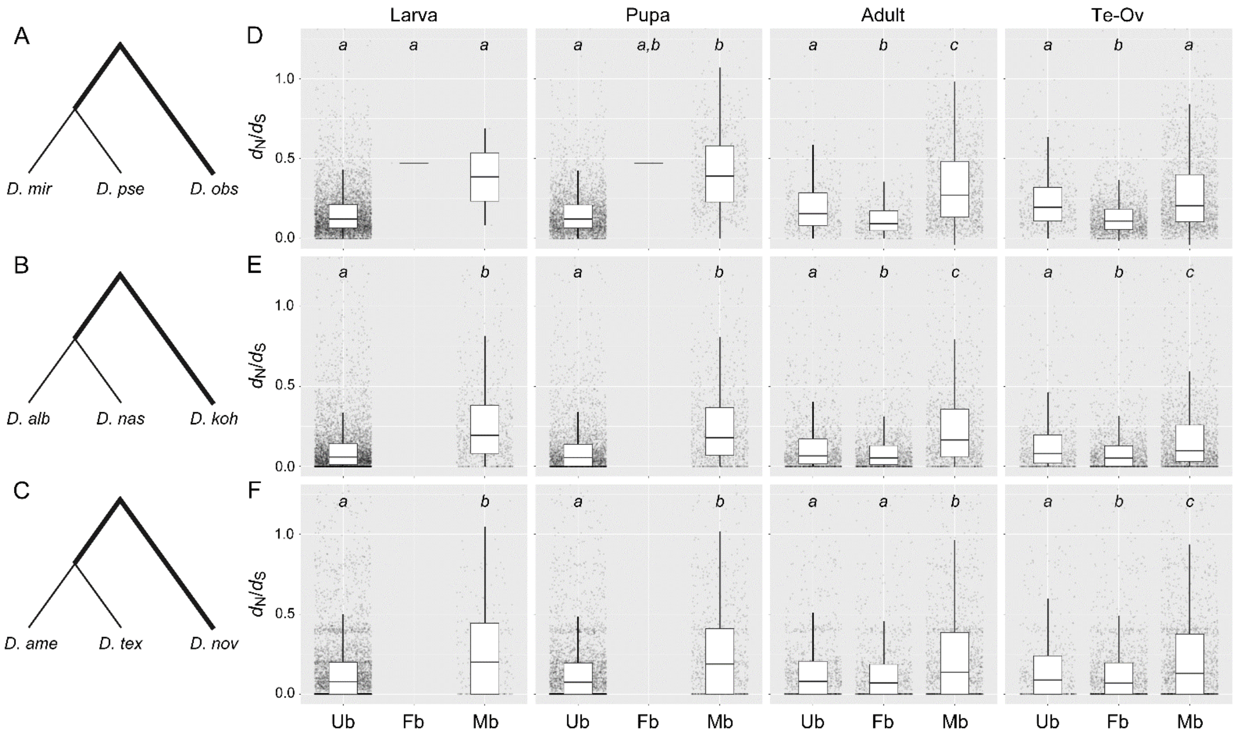

Figure S6. Relationship between functional constraint and sex-biased expression in the lineages of (A and D) *Drosophila miranda* and *D. pseudoobscura*, (B and E) *D. albomicans* and *D. nasuta*, and (C and F) *D. americana* and *D. texana*. The ratio of the numbers nonsynonymous to synonymous substitutions per site was computed in the lineage of (A) *D. obscura*, (B) *D. kohkoa*, and (C) *D. novamexicana* as shown in bold, and plotted with three categories for sex-biased expression in larvae, pupae, adults, and gonads (D-F). Ub, shared unbiased genes in the trio (e.g., *D. miranda*, *D. pseudoobscura*, and *D. obscura* in D); Fb, shared female-biased genes in the trio; Mb, shared male-biased genes in the trio. Differences between categories were tested by a Mann–Whitney *U* test with correction for multiple testing by the Benjamini-Hochberg method (Benjamini & Hochberg, 1995). The same letter in italics indicates $Q \geq 0.05$, whereas different letters indicate $Q < 0.05$ for comparisons between categories.

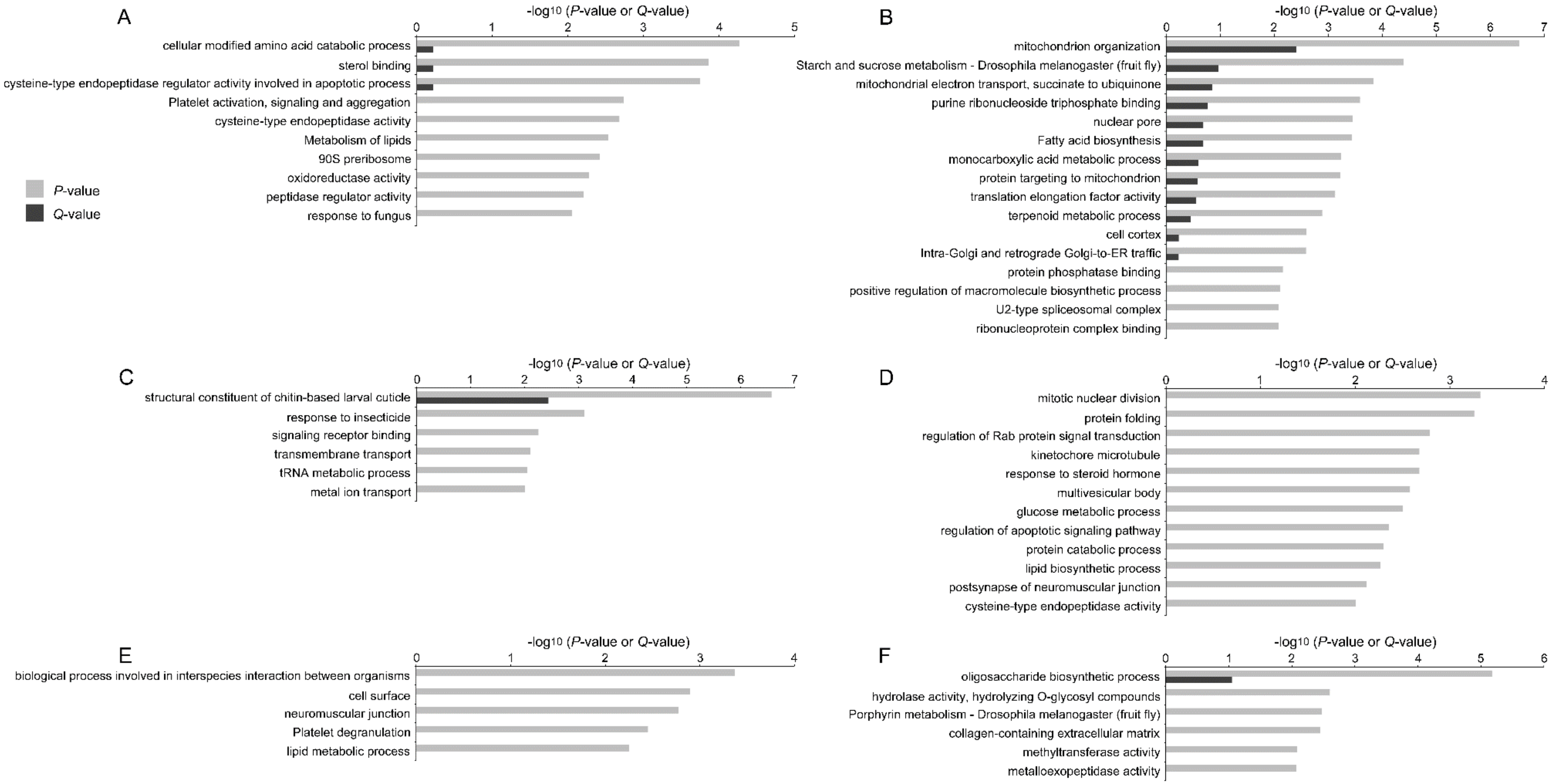

Figure S7. Terms enriched in species-specific sex-biased genes on the neo-sex chromosomes in (A and B) the *Drosophila miranda*, (C and D) the *D. albomicans*, and (E and F) the *D. americana* larvae. The genes are unbiased genes in the other two closely-related species of each trio. (A, C, and E) Species-specific female-biased genes. (B, D, and F) Species-specific male-biased genes. Only top 20 terms (*P*-value < 0.01) are shown at maximum. Metascape software (Zhou et al., 2019) was used for the analysis.

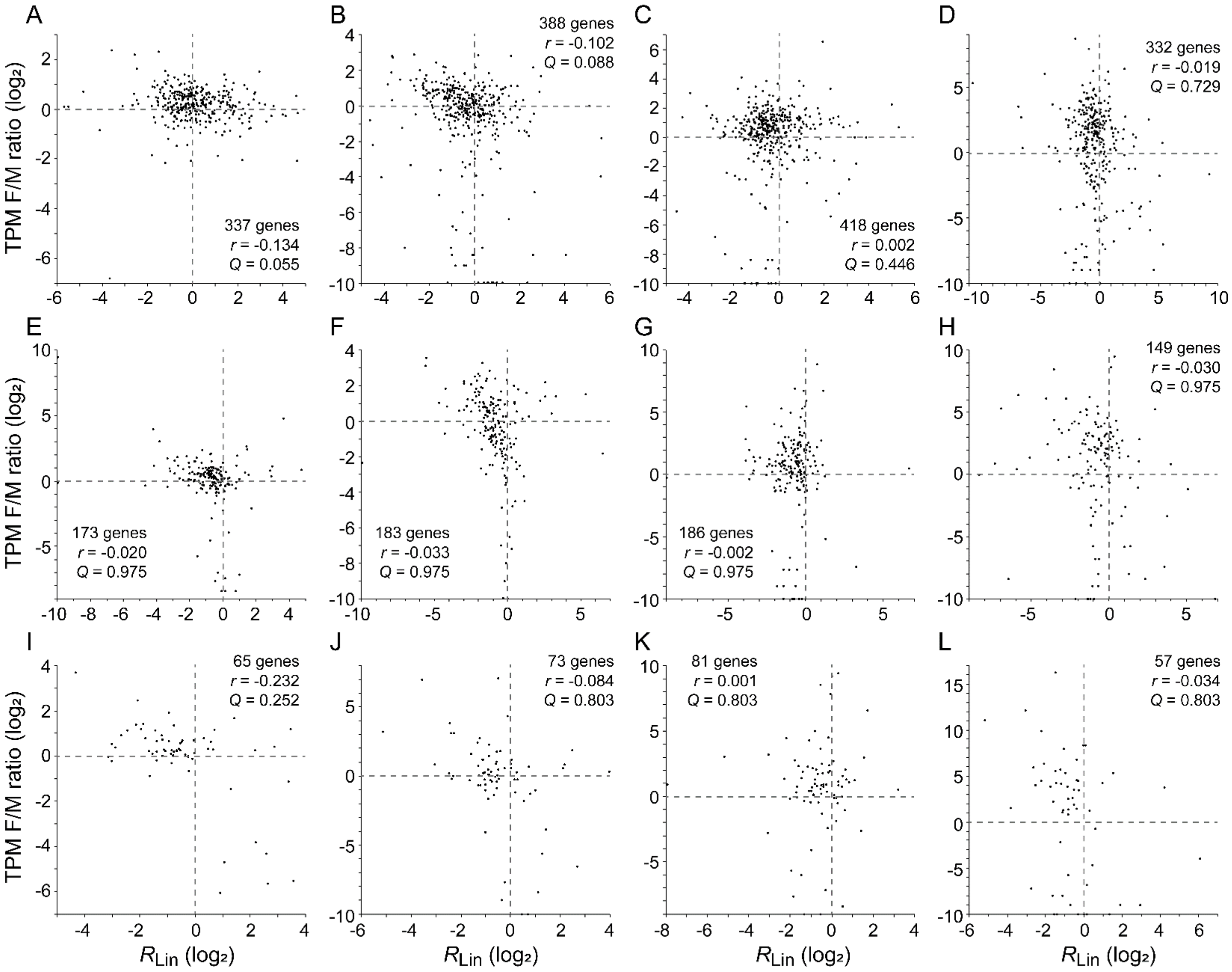
A
337 genes
r = -0.134
Q = 0.055
B
388 genes
r = -0.102
Q = 0.088
C
418 genes
r = 0.002
Q = 0.446
D
332 genes
r = -0.019
Q = 0.729
E
173 genes
r = -0.020
Q = 0.975
F
183 genes
r = -0.033
Q = 0.975
G
186 genes
r = -0.002
Q = 0.975
H
149 genes
r = -0.030
Q = 0.975
I
65 genes
r = -0.232
Q = 0.252
J
73 genes
r = -0.084
Q = 0.803
K
81 genes
r = 0.001
Q = 0.803
L
57 genes
r = -0.034
Q = 0.803
TPM F/M ratio (log2)
RLin (log2)

Figure S8. Relationship between dosage compensation and sex-biased expression of neo-X-linked genes in (A-D) *D. miranda*, (E-H) *D. albomicans*, and (I-L) *D. americana*. Samples for examining the relationship are as follows: (A, E, and I) larvae, (B, F, and J) pupae, (C, G, and K) adults, (D, H, and L) gonads. R_{Lin} (Lin et al., 2012) was used as an index showing the extent of dosage compensation for each gene. The value of 1 indicates perfect dosage compensation whereas the value is expected to become 0.5 if there is no dosage compensation. Grey broken lines represent the R_{Lin} value of 1 for the X axis and the ratio of female to male TPM value of 1 for the Y axis. The genes that are pseudogenized on the neo-Y and have orthologs on the same Muller element in the closely related species (e.g., *D. pseudoobscura* and *D. obscura* for *D. miranda*) and show the TPM value of ≥1 in at least either female or male tissues were analyzed. The number of genes analyzed and correlation coefficient (*r*) were shown in the margin of each plot. Significance of the correlation was tested by a t-test with correction for multiple testing by the Benjamini-Hochberg method (Benjamini & Hochberg, 1995) and shown by *Q*-value.

REFERENCES

Bachtrog, D. & Charlesworth, B. (2002). Reduced adaptation of a non-recombining neo-Y chromosome. *Nature*, 416, 323-326.

Benjamini, H. & Hochberg, Y. (1995). Controlling the false discovery rate: a practical and powerful approach to multiple testing. *J R Statist Soc B*, 57, 289-300.

Gao, J. J., Watabe, H. A., Aotsuka, T., Pang, J. F., & Zhang, Y. P. (2007). Molecular phylogeny of the Drosophila obscura species group, with emphasis on the Old World species. *BMC Evol Biol*, 7, 87.

Lin, F., Xing, K., Zhang, J., & He, X. (2012). Expression reduction in mammalian X chromosome evolution refutes Ohno's hypothesis of dosage compensation. *Proc Natl Acad Sci USA*, 109, 11752-11757.

Morales-Hojas, R., Reis, M., Vieira, C. P., & Vieira, J. (2011). Resolving the phylogenetic relationships and evolutionary history of the Drosophila virilis group using multilocus data. *Mol Phylogenet Evol*, 60, 249-258.

Nagaraja, Nagaraju, J., & Ranganath, H. A. (2004). Molecular phylogeny of the nasuta subgroup of Drosophila based on 12S rRNA, 16S rRNA and CoI mitochondrial genes, RAPD and ISSR polymorphisms. *Genes Genet Syst*, 79, 293-299.

Satomura, K. & Tamura, K. (2016). Ancient male recombination shaped genetic diversity of neo-Y chromosome in *Drosophila albomicans*. *Mol Biol Evol.*, 33, 367-374.

Vieira, C. P., Coelho, P. A., & Vieira, J. (2003). Inferences on the evolutionary history of the *Drosophila americana* polymorphic X/4 fusion from patterns of polymorphism at the X-linked paralytic and elav genes. *Genetics*, 164, 1459-1469.

Zhou, Y., Zhou, B., Pache, L., Chang, M., Khodabakhshi, A. H., Tanaseichuk, O., Benner, C., & Chanda, S. K. (2019). Metascape provides a biologist-oriented resource for the analysis of systems-level datasets. *Nat Commun*, 10, 1523.
